# Acute Renal Failure with Overdose of Labetalol: Special Considerations in Management

**DOI:** 10.7759/cureus.1657

**Published:** 2017-09-06

**Authors:** Praveen Guturu

**Affiliations:** 1 Internal Medicine, University of Texas Medical Branch

**Keywords:** labetalol, acute renal failure

## Abstract

We report a case of acute renal failure in a patient with labetalol overdose, discuss the possible pathogenesis, and highlight special considerations in the management of labetalol overdose as compared with other beta-blocking agent overdoses.

## Introduction

Acute renal failure is uncommon in pure beta adrenergic blocker toxicity, but labetalol, with its alpha blockade, can lead to complex hemodynamic changes and can cause acute renal failure at toxic levels. Understanding the pathogenesis of renal impairment with combined alpha and beta blocker overdose will help us to manage the complications more effectively.

## Case presentation

A 38-year-old female patient was transferred to our tertiary care center from another hospital with an overdose of approximately 12-14 grams of labetalol with alcohol. Her past medical history is unremarkable. In a suicidal attempt, she ingested the labetalol prescribed to her husband one-two hours prior to presenting to the hospital.

At the time of presentation at the outside hospital, she was hypotensive at 54/33 mm of Hg and her heart rate was approximately 60 beats/minute. Her physical exam was within normal limits and her mentation was intact. Serum creatinine was 1.1 mg/dl (83.3 µmol/L), CO_2_ was low at 18 mmol/L, acetaminophen/salicylate was undetectable, creatinine phosphokinase levels were normal, and serum lactate and serum labetalol levels were not obtained. 

Initial therapy at the outside hospital included fluid boluses, glucagon, calcium gluconate, and dopamine infusion. The patient remained hypotensive and anuric, so after consultation with the poison center, norepinephrine was added. After three-four hours, her urine output and blood pressure improved, so norepinephrine was discontinued, and she was transferred to our center.

Upon arrival at our center, she was six hours post-ingestion and was on dopamine only. Her blood pressure was 84/37; she remained alert and was in no acute distress. Her creatinine was 2.63 mg/dl (200 µmol/L). On arrival, dopamine was switched to norepinephrine and eventually discontinued, as her urine output and blood pressure remained stable and the abdominal ultrasound was within normal limits. Over the next few days, her urine output improved, but her serum creatinine continued rising, reaching 5.4 mg/dl (411.8 µmol/L) by day five.

Causes such as myoglobinuria and interstitial nephritis were excluded in our patient by urinalysis, which showed muddy brown casts. Acute tubular necrosis is the possible diagnosis but as the patient was asymptomatic and non-oliguric, a renal biopsy was not performed. She was asymptomatic throughout her stay and maintained good urine output. She was managed conservatively, needing dialysis, and was discharged once her serum creatinine started decreasing. She did not keep her two-week follow-up appointment.

## Discussion

In 2006, the US poison center annual report included 9041 cases of single-agent poisoning involving beta-blocking agents and the outcome was fatal in four cases [1]. Prolonged hypotension is reported after large doses of ingestion but renal failure is not very common. Further, a reduction in the severity of acute renal failure in rats by beta-adrenergic blockade is reported [2].

Acute renal failure has been reported twice in the past with an overdose of labetalol, a selective α 1-antagonist and a non-selective β-antagonist [3-4]. Acute renal failure with an overdose of beta-blocking agents is not very common and a reduction in the severity of acute renal failure with the administration of beta blockers is demonstrated in experimental settings in animal studies [2].

With prolonged hypotension and ischemic acute renal failure, it is postulated that the kidneys lose their ability of auto-regulation via the renin angiotensin system; however, in beta-blocker overdose, there is uninterrupted alpha stimulation, leading to efferent arteriole vasoconstriction, and, so, maintenance of intraglomerular pressures, which helps to maintain the glomerular filtration rate and avoid uremia [5]. But, with cases involving labetalol overdose, the alpha receptor antagonism will lead to efferent arteriole vasodilatation and a fall in the intraglomerular pressure; consequently, there is a reduction in the glomerular filtration rate. Similar hemodynamic changes have been proposed as the basis of acute renal failure in humans [6].

According to current evidence, glucagon is the first line of therapy [7]. Glucagon is thought to activate adenylate cyclase in cardiac tissue by directly stimulating a G protein on the β-receptor complex. Calcium, insulin, glucose, and catecholamines can be used for supportive treatment, as needed. Unlike pure beta adrenergic blocking agents, the alpha-blocking properties of labetalol add complexity to management and phosphodiesterase inhibitors like amrinone have proven to be helpful in cases of labetalol overdose [8].

The use of dopamine in our patient might have also contributed to the loss of filtrating pressures by its vasodilating effects. Although we expect dopamine to stimulate alpha-1 receptors at high doses, causing vasoconstriction, its use with glucagon is associated with detrimental effects largely due to their opposing actions on various vascular beds, which may result in an unpredictable maldistribution of blood in microcirculation and impaired perfusion of vital organs [9].

Measuring the levels of the offending drug might help to confirm the diagnosis; however, drug levels are not performed locally in most clinical laboratories. This might cause a delay in specimen handling and in reporting the results back to the clinician, and clinical symptoms might not necessarily correlate to the drug level. Therefore, the management and treatment of a beta blocker overdose should be instituted based on the clinical presentation and history of ingestion [10].

## Conclusions

Acute renal failure is uncommon with pure beta blocker toxicity but overdose with a combined alpha and beta blocker like labetalol can lead to acute renal failure, as presented here. It is crucial to recognize this uncommon presentation of renal failure associated with the overdose of labetalol because prompt recognition is necessary to initiate the treatment with supportive therapy and glucagon and prevent the worsening of renal failure.
